# Monitoring and Efficiency in Governance: A Measure for Sustainability in the Islamic Banking Industry

**DOI:** 10.3389/fpsyg.2022.884532

**Published:** 2022-06-29

**Authors:** Muhammad Awais, Naeem Ullah, Numair Ahmad Sulehri, Mohamed Asmy bin Mohd Thas Thaker, Muhammad Mohsin

**Affiliations:** ^1^Department of Economics and Finance, Faculty of Management Sciences, Foundation University School of Science and Technology, Rawalpindi, Pakistan; ^2^Department of Business Administration, Faculty of Management Sciences, Foundation University School of Science and Technology, Rawalpind, Pakistan; ^3^Department of Economics, International Islamic University Malaysia (IIUM), Kuala Lumpur, Malaysia; ^4^Riphah International University, Rawalpindi, Pakistan

**Keywords:** governance, sustainability, Islamic banking, Pakistan, Malaysia

## Abstract

Corporate governance is a set of rules, regulations, procedures, processes, and practices through which an organization is controlled and directed. The present study aimed to examine the monitoring methods used in Islamic banking, including standardized measures for better performance, an individual’s aptitude towards Islamic financial markets, risk propensity, and the level of efficiency of the Islamic banking industry in Pakistan and Malaysia. There is room to improve monitoring systems for Islamic banking operations and standardized measures could improve efficiency, leading to more sustainable performance. The study uses a self-developed semi-structured scale based on literature and expert interviews, after content and context validity to gain a wide range of diverse information. In Pakistan and Malaysia, individuals’ perceptions are different because of differences in the banking environment and preferences. Eventually, the Islamic banking growth rate may differ in Pakistan and Malaysia. Thus, there should be regular monitoring to improve banking performance. Similarly, standardized measures for Islamic banking operations and governance performance in Pakistan and Malaysia will result in more sustainable performance. The antecedents of Islamic corporate governance could be improved to enhance banking performance, which helps individuals make decisions based on available product information. The business growth of the banking industry is based on convenient monitoring policies, standardized performance measures, and, most importantly, excellent corporate governance mechanisms. Improved monitoring measures will further enhance these business operations.

## Introduction

Private monitoring consists of two types, the first is external, which opines that monitoring is mainly a task of institutional investors (Mubeen et al., 2021a; Ge et al., 2022; Liu et al., 2022). The second is internal monitoring mechanisms, comprised of CEO and chairman separation of designation, independent board members, and an audit committee (Aqeel et al., 2021, 2022; Aman et al., 2022; Rahmat et al., 2022; Yao et al., 2022). The chief is on the Board of Directors (BOD) for the provision of the monitoring, controlling, and advisory commitments of the firm (Abbas et al., 2020; Mamirkulova et al., 2020; Mubeen et al., 2021b; Liu et al., 2022). They can build or deteriorate corporate governance (Balachandran and Williams, 2018). When BOD planning goes unexpectedly the company might enter into a state of bankruptcy (Mubeen et al., 2020; Wang et al., 2021; Fu, 2022). The BOD has the authority to fire a CEO if they do not comply with desires and expectations. Usually, the BOD fires an incompetent CEO based on soft information (Laux, 2008), which ideally results in the better performance of the firm (Khazaie et al., 2021; Paulson et al., 2021; Yoosefi Lebni et al., 2021; Li et al., 2022; Mamirkulova and Mi, 2022). BODs typically have two differing views: (1) Board capture means that the CEO can exert pressure on inside directors based on self-interest, which only maximizes the CEO’s well-being. (2) Conversely, the CEO might select internal directors based on maximizing the shareholders’ wealth because they are astute and connoisseurs of the internal affairs of a firm (Malik and Majeed, 2017; Irfan and Ali, 2019; Naveed et al., 2019; Shahzad and Malik, 2019; Ashraf et al., 2020).

As per Mollah and Zaman (2015), conventional banks (CBs) hire more internal CEOs than Islamic Banks (IBs; Abbas et al., 2019a, 2019b). The board of IBs takes more independent decisions than CBs. In non-Islamic boards, members tend to be more concerned with profit generation. Contrary to this, the independent directors of IBs are linked with a decline in the performance of the firm (Badar and Irfan, 2018; Ali, 2020; Ali et al., 2021; Zhu et al., 2021). The presence of multi-layer corporate governance in Islamic banks helps them to perform better than conventional banks (Elgattani and Hussainey, 2020). This also assists in the protection of shareholders. Board structure and CEO power also affect the performance of the firm (NeJhaddadgar et al., 2020; Azizi et al., 2021; Maqsood et al., 2021).

Banks with high individualism tend to have higher incentives but banks with high power hold less leverage (Zhang et al., 2012; Asad et al., 2017; Ahmad et al., 2021). As per Kutubi et al. (2018), board busyness has a U-shaped relationship with a bank’s risk and performance. The Brobdingnagian amount of losses reported are indicative of the performance of conventional banks and Islamic banks, and need to be analyzed, classified, reckoned, and identified for them to be managed effectively and efficiently.

As mentioned above, the better the audit committee the better the corporate governance (Abbas et al., 2019c, 2020; Asad et al., 2020). The BOD and management usually nominate audit committees with external directors to provide impartial and unbiased decisions to better the company (Haldar and Raithatha, 2017). Their main function is to oversee issues and help the BOD with financial management. One of its chief functions is to act as a bridge between the external auditor and company matters.

Since the inauguration of the Sarbanes Oxley Act (2002), there has been an enormous amount of external directors appointed to ensure transparency. External directors have more material effects than internal ones. Intense monitoring forces the CEO to share necessary information with the BOD. External directors do not face lawsuits when the company requires accounting restatements but there is a labor market penalty. Directors who play a vital role in different companies are busy and might attenuate governance to enhance CEO compensation, which is outright exploitation of a firm’s performance. On the other hand, busy directors are positively associated with new public firms.

CEO compensation plays a very important role in driving CSR (Peng, 2020) and controlling earning management (Park, 2017). Additionally, higher analyst coverage in terms of financial statements results in higher firm transparency and lower earnings management. Some authors (Winecoff, 2017; Thiemann et al., 2018; Tchamyou, 2021) outline that present data on bank governance is not sufficient to articulate a link between governance and global financial crises. Banks with shareholder-friendly boards face declining performance in global financial crises. Culture also plays a role in risk-taking and creates measurement complexities (Dubey et al., 2017). Shareholder wealth maximizes when in the terms of banks have higher inside shareholders, i.e., CEO and lesser franchise values.

This paper specifically targets Pakistan as a research medium and analyses to what extent Islamic finance interlinks with corporate governance. To find evidence of the links between Islamic laws and corporate governance, we examined examples from the banking and finance industries.

Although all the factors mentioned above have already been studied, they have not been considered in relation to the context of Pakistan and Pakistani culture. In the same way, there are similarities in performing operations, actions, and approaches to mitigating problems between conventional boards and Shariah Supervisory Boards (SSBs). Islamic banks perform their actions within the Islamic framework (Ahmed, 2011), but in Pakistan, banks tend to follow non-Islamic corporate governance regulations instead of the Islamic perspective. This does not signify that one of these structures is better than the other, as it depends upon culture, context, and religious beliefs. This paper substantiates that the “Anglo-Saxon model” (a capitalist model) of economics implements the stakeholder theory (a theory of organizational management and business ethics that accounts for numerous communities wedged by commercial entities like workers, dealers, native groups, creditors, and others) whilst considering how this theory is inherent and built into the framework of Islam. According to the Islamic framework, a company is culpable and accountable to society and stakeholders.

To critically analyze the organization of economic cooperation and development (OECD) within the Islamic context we found that there are similarities between conventional boards and SSB. When considering corporate governance in Pakistan it is unjust to compare it with the Anglo-Saxon model or structures in developed countries as, even though it is present in the constitution, corporate governance in Pakistan, is still in its initial stages. Differences, therefore, exist due to the novel structure of Pakistan and the old structures used in developed countries like the UK. For example, concepts of ownership structure, laws, and regulations, etc. To date, few codes have been extended in Pakistan regarding corporate governance, and the few that have tend to abide by the Anglo-Saxon model structure rather than Islamic.

This research is necessary to galvanize better corporate governance structures in Pakistan where, to date, there has been a lack of disclosure, transparency, and accountability, and alternative structures of corporate governance are needed. The present study explores the interlinking between Islamic laws and corporate governance in Pakistan, using examples from the banking and finance industries in both Pakistan and Malaysia.

## Literature Review

The qualities and adeptness of corporate governance and audit committees along with previous literature inform this paper’s exploration of corporate governance and the roles and responsibilities of audit committees. Better corporate governance means that performance is also better because with better corporate governance shareholders and stakeholders are more satisfied. This eventually helps the firm to maximize its profits (Ramli and Ramli, 2016).

Discrepancies between actual and desired corporate governance are a vast issue (Mahrani and Soewarno, 2018; Jia et al., 2019). For instance, from the disclosure of financial reporting to audit committee oversight, there have been colossal discrepancies in economic downturns (Rahim et al., 2015). To scrutinize this we garnered facts about the composition and communication of audit committees.

Non-financial companies typically have high amounts of debt to pay and their corporate governance is also impaired (Mubeen et al., 2020; Nawaz et al., 2020). Maximum ownership usually resides within families, meaning the decisions of the firm serve their personal interests (Abbas et al., 2019d; Aman et al., 2019; Zhou et al., 2022), ultimately this can undermine the interests of minority shareholders. In contrast, the sole purpose of Shariah-compliant firms (SCFs) is that they are governed by the sovereign power of “Allah.” This is a driving force for SCFs to act ethically and as per the stipulated and articulated Islamic principles (Imamah et al., 2019). This requires self-monitoring because, at the end of the day, managers are solely accountable to the power of accountable to Allah (SWT).

The effect of religion on economy and decision-making has been thoroughly researched by Max Weber. This research compared two branches of Christianity, exploring Protestant beliefs and Catholicism. According to this research, the preaching of Catholics is associated with the growth of the economy. In the same way, some studies (Bello and Bello, 2017; Elnahas et al., 2017; Belligni, 2019) show a direct and positive relationship between religion and economic boom. A number of studies (Li et al., 2019; Davis and Renzetti, 2021) have asserted that companies founded by religious people take less risk. In the same way, companies that reside in religious areas tend to take fewer risks. Nevertheless, companies established in areas where activities such as gambling take place tended to be innovative (Jang et al., 2017). In the same way, religious employees tend to disallow and have a strong anathema to manipulating accounts, value-destroying behavior, and aggressive accruals.

Islamic finance has been in accretion ever since the UK announced that it would provide Islamic bonds. These bonds were floated in places such as Singapore and Hong Kong. These assets doubled between 2003 to 2013. This colossal increase in Islamic finance warrants further academic research, which is chiefly possible due to data availability. The availability of data, however, can result in a blinkered perspective, as one cannot see the full picture of Islamic finance beyond banking.

A study of 21 Gulf countries shows that Shariah Supervisory Boards (SSBs) disclosed more social activities than annual reports (Mallin et al., 2014). Social and ethical activity creates goodwill and is as significant as the financial performance of the firm. Nonetheless, empirical findings assert that there is a neutral relationship between these two. This is due to profit and loss sharing, as banks may pay zakat on behalf of their customers and Qard Hassan may also be provided to benefit society. According to slack resource theory, the relationship between CSR-FP is the inverse of “FP-CSR.” This is substantiated by firms that have higher financial performance, which tend to spend a higher amount of slack resources on CSR activities. This will assist in enabling better social performance management.

Based on this social performance management, Islamic banks have expanded their assets to around US$1.3 trillion, indicating that it is a fast-growing industry (Neifar and Jarboui, 2018). This shows its importance in the international financial system. Islamic banks have a domineering effect on the entire Islamic financial industry due to their high amount of assets. Thus, the disclosure of operational risk is cardinal to Islamic banking operations. This also assists in ameliorating the information asymmetry in the firm.

Alhammadi et al. (2020) state that the biggest risk for Islamic Financial Institutions (IFI) is margin risk, followed by operational risk. The former risk is instigated due to poor human resource strategies and poor systematic legislation.

Comprehensive tests were undertaken to explore how Islamic finance is being proliferated all around the globe. In particular, we explored how it has been used by international governments and firms, especially in second-world countries (Alzahrani, 2019). Even though Islamic (academic) research is often skeptical about following and accepting Islamic finance as a subject matter of empirical and theoretical analysis, the establishment of a journal of corporate finance will certainly highlight practicalities for corporate managers and demonstrate the functioning and well-developed state of Islamic finance.

Islamic banks are 4 percentage points better off than conventional banks in terms of being cost-efficient. However, they are 17 percent less profit efficient (Safiullah and Shamsuddin, 2019). Studies have shown that the comparison of conventional and Islamic banks has been a topic under discussion in recent years. Ali and Azmi (2016) argue that conventional banks should be supplanted by Islamic banks outright because conventional banks show no more or less recuperation in financial crises, whilst Islamic banks exhibit more resilience during the interim. However, religion is the sole catalyst of innovation and risk-taking decisions (Torlak et al., 2021). Financial statements are a judgmental elements (based on which you can take investment decisions) as religious people sometimes morally object to the account’s manipulation (window dressing).

Socially responsible investment (SRI) is an important aspect of Islamic banking. SRI has become important in the last few years and these types of investments have certainly increased by 9% (Azmi et al., 2019). Islamic finance has increased during these years but is also gradually decreased in some respects. This happened because Islamic fund managers had an advantage by screening Shariah-compliant equity funds. SRI disallows un-Islamic investments, for instance, investment in weapons and gambling, etc. This puts limitations on Shariah-compliant assets.

## Methodology

This study used an interview-based multi-case study technique (Eisenhardt, 1989; Eisenhardt and Graebner, 2007), in which shreds of evidence were collected *via* semi-structured deliberations to gain information and statistics apropos the sustainability and efficiency of the governance system of banks. In this paper, a qualitative technique was used to receive answers from interviewees. No quantifiable information was provided before the study and no evaluation of prospective responses was shared (Aqeel et al., 2021; Farzadfar et al., 2022). The numerous cases offer insights into central businesses through the close inspection of themes and proof. The case study decorum (Haddock-Millar et al., 2016) is outlined in Table 1.

**Table 1:** Case study protocol.

Steps of the case study
1. To ascertain the focus and scope of the research.
2. Identification of discrete affluence to become ‘multiple cases’.
3. Assisting the development of the research questions.
4. To find factual tools for the research and etiquettes of the study, gaining qualitative data by amassing semi-structured deliberations and focus groups.
5. To find ‘fitting’ respondents: a straight and uniform piece of the case studies with familiarity with conservational and HRM/growth.
6. The data gathering took place in Pakistan in August 2021.
7. Data analysis: privately surrounded by the case at the solo subsidiary level – Pakistan.
8. Progression of prime themes – Pakistan.
9. The data gathering took place in Malaysia in August 2021.
10. Data analysis: privately surrounded by the case at the solo subsidiary level – Malaysia.
11. Progression of prime themes – Malaysia.
12. Cross-case analysis – Pakistan and Malaysia.
13. Contrast of literature: identification of similitudes and inconsistencies.
14. Attaining closure: literature and data accumulated.
15. Dissemination: development of the article.

The discussions related to the theme of the study – the monitoring and efficiency in governance systems – and measure sustainability in the banking sector. This study uncovered diverse angles that were situation-specific, allowing for a proportional analysis of approaches to and the practice of Islamic Capitalism towards enhancing the growth of Islamic Finance.

Interviewees included Islamic financial advisors, monitoring experts, and policymakers (governance) from the banking sectors of Pakistan and Malaysia. Semi-structured meetings were used to direct discussions and gain in-depth information. In total, the study arranged 26 interviews – 18 frontal and 8 through an open-ended scale (Table 2), however, only 21 participants provided all-inclusive and sound replies as per the prerequisite of the study.

**Table 2:** Plaintiffs of the discussions.

Job role	Pakistan	Malaysia
*Category*		
Islamic financial advisors	8	2
Monitoring experts	5	2
Policymakers (governance) from banking sectors	7	2
Total participants	20	6
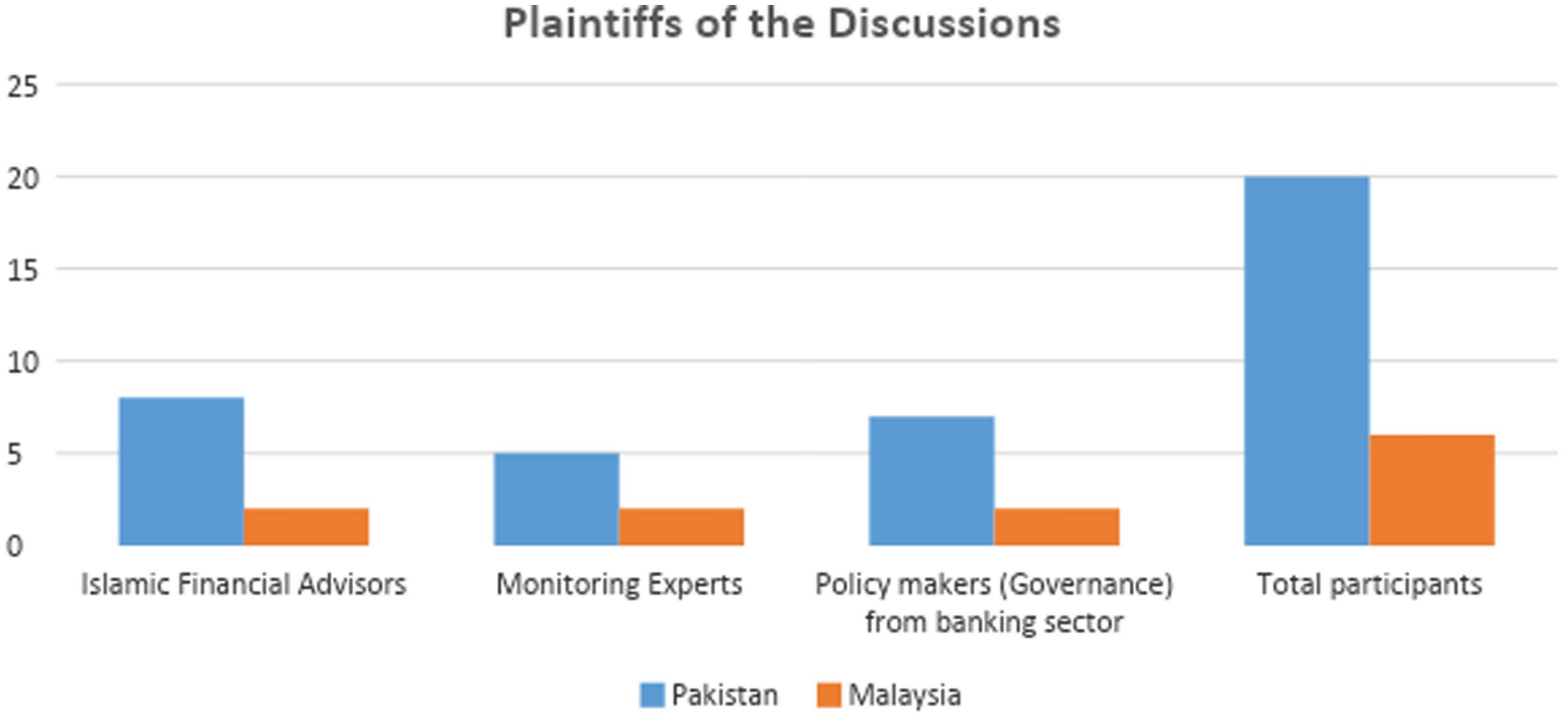		

Table 2: Job Roles. This table speculates that whole contributors have excessive data concerning Shariah-based speculation and tradeoff practices and processes, and the wide-ranging ups and downs of the market.

The focal confab questions (questions asked during an interview) were taken from literature on the extent of Islamic monitoring and governance systems and the sustainability of the Islamic banking sector. The confab questions are provided in Table 3.

**Table 3:** Confab protocol and probes.

***Confab protocol***
Present the interviewer(s) and interviewee(s)
Plan the complete study
Outline the fortitude of the study, accompanied by penalty area and objectives
Treatise on the probable outcomes of the study, including moral issues and gaining permissions
Draught edifice of the confab/focus group
***Themes of the study and obvious probes***
***Islamic financial advisor’s points of view on current situations***
1. What role does your advice play in changing policies towards monitoring and governance structure?
2. How your advice may play a role in the sustainability of the banking sector?
3. How would you describe the existing situation of the Islamic banking sector?
4. What are the significant effects of current situations on the monetary condition of the banking sector?
5. Do you think investment choices based on Shariah are life-shaping verdicts of the Muslim investors?
6. Can you please describe the most noteworthy divine perspicacity of Muslim investors and practitioners at the time of decisions?
***Monitoring expert’s points of view on existing policies***
1. What are the leading features for monitoring in the banking sector?
2. What is the role of monitoring towards sustainability in Islamic banking?
3. How is monitoring in Islamic banking different from conventional approaches?
***Point of view of Policymakers (governance) from Islamic banking sectors on existing conditions***
1. How might you set the benchmark for governance structure in an Islamic banking setup?
2. Is there any benchmark for Islamic corporate governance?
3. Have you completely identified the antecedents of Islamic corporate governance?
4. Why is investment based on Shariah good in Pakistan/Malaysia?
5. How do individuals in the banking sector come to an understanding about investment and exchange decisions based on Shariah approaches to banking?
6. What part do you play in persuading individuals to accept Shariah-based governing principles?
7. What is the impact of an individual’s psychological approach on the market?
***Sensitivity of the Islamic banking sector***
1. How subtle is the Islamic banking sector of Pakistan/Malaysia?
2. Which level of efficiency (weak, semi-strong, and strong) exists in the financial market of Pakistan/Malaysia?
***The risk-taking propensity of individuals***
1. Do you think individuals are not concerned about Islamic Corporate Governance? If “Yes,” then how might you inspire them to adopt Islamic Corporate Governance?
2. Are conventional individuals more likely to take high risks or low risks?
***About you***
1. What are the main factors that influence you at the time of taking decisions?
2. What do you perceive as the key foundations of a rational Shariah-based governance structure?

The confab involved a dialogue on the Islamic monitoring system in the market, the life-shaping decisions of Muslim investors through Shariah-based governance practices, executives and practitioner psychosomatic intuitions, the sensitivity of the banking sector, and the daring propensity of persons in the marketplace. The confabs then explored the areas discussed in the literature, on the practice of Shariah-based approaches to monitoring and governance. The frontal confabs (forward discussions) lasted between 30 and 50 min and up to 60 min for each individual.

## Findings and Discussion

### Islamic Financial Advisors

Corporate governance is a set of rules that an organization follows to mitigate fraudulent activities. These are the internal and external affairs of an organization and environmental factors that influence the decisions of an enterprise. To assimilate corporate governance in terms of financial performance, we first need to shed light upon the strengths of corporate governance (Liu et al., 2022). Corporate governance strength is underscored by corporate governance disclosure, and the present study provides advice for policy setting as per these standards.

Individuals and investors have different individual perceptions of the existing situation in the banking sector. The implementation of corporate governance varies across borders. This means that corporate governance needs to be implemented differently in different regions.

Despite the six principles of the Organization of Economic Cooperation and Development (OECD) there are still differences in perception across different countries, for example in the USA and Saudi Arabia. The establishment of corporate governance was initiated in the USA and then it shifted to the United Kingdom. It was initially an Anglo-American process. The exact definition of corporate governance had not been previously formulated. Therefore, researchers usually related agency cost theory with corporate governance and consider Anglo-American and continental European corporate governance comparable. The use of various models may give diverse returns in various circumstances. Moreover, most Muslims invest as per the religious teachings on investment because they want to secure their life after death, and people often think that investment as per Shariah principles will efficiently change their life.

### Monitoring Experts

Corporate governance gains new dimensions when based on the Islamic rules of the sovereignty of Allah Almighty and with the belief that Prophet Muhammad S.A.W is the last messenger. Islam creates a context of reference and rules from the perspective of the teachings of the Quran and Sunnah. The amalgamation started after the Sarbanes Oxley act when Tyco international showed its dark colors with fraudulent activities. In the conventional banking sector, interest rates, yield curves, and net interest margins for the state of the economy are gaged by GDP growth, employment growth, inflation, and currency fluctuations, which are considered the main aspects in terms of monitoring. By contrast, in Islamic banking, employment growth, inflation, and currency fluctuations are commonly monitored.

Islam and corporate governance are two distinct areas. The former is related to religious perspective and the latter is related to organizational orientation. This provides a new dimension to the corporate world. Although there has been a preponderance of studies that show agency costs are mitigated with better corporate governance, according to the Islamic perspective, the agency cost seems to diminish. This is theoretical and has not been empirically proven to date. Although the presence of corporate governance is supposed to benefit shareholders through procedural rules and principles, the companies still faced a huge disaster in the 1990s and early 2000s due to the high salaries of strategic managers (Erturk et al., 2004). It is important that monitoring is undertaken on a timely and regular basis, meaning the controlling authority can track planning and the use of the resources. It offers decision-makers an approach to sustainably planning for ventures and future actions. Moreover, monitoring criteria are usually based on the operational activities of the organization, but most of the determinants are common in all the subjects.

### Policymakers (Governance) From Islamic Banking Sectors

Corporate governance exists on two levels; it is either poor or good. Traditional wisdom is associated with corporate governance. It is engendered in such a way that explains the cause and effect of corporate governance with competition in firms and different industries operating under a single economy.

As per the governance methods, corporate governance has different definitions according to different authors. However, we can define corporate governance as an outright set of responsibilities that are shared among a board of directors to shield the rights of shareholders and stakeholders (Robertson et al., 2013). With its definition come the ties of Islam within Islamic institutions. For example, Islamic principles-based banks. Their operationalizing is different from conventional banks because of the addition of one religious element, EG Islam in terms of the Sunnah, Shariah, Quran, and teachings of the Prophet Muhammad (S.A.W). Moreover, it is extremely difficult to explore the complete antecedents of Islamic corporate governance.

In response to the many determinants of corporate governance, let us lucubrate the amalgamation of Islam with corporate governance and its determinants. Keeping corporate governance under, Islamic laws, principles, Shariah, Quran, and Sunnah are beneficial for a firm, enabling it to have a better grip on decision making. Islam has its rulings, which are further divided into worshipping (Allah is the only sovereign power) and Muamlaat, also known as mutual dealings between two parties. This helps to mitigate agency costs that are solely based upon agent and principal self-interests. Muamlaat has further polythetic characteristics known as the “Doctrine of Universal Permissibility,” which means that the two parties can enter a contract without having to sacrifice the Shariah principles. Furthermore, the implementation of ICG means that the views of traditionalists and the views of modernists have to be taken into account for the sake of implementation of ICG. Traditionalists have antithetical views concerning modernists. Traditionalists make use of Ijtihad to interpret the teachings of the Quran and Sunnah in the best way possible. The collaboration of Islamic scholars and financial secular experts would push technical advancements in banking.

Western models of capitalism are vastly different from Islamic capitalism because Islamic capitalism is based upon Islamic finance and the rulings of the Quran and Sunnah. As soon as Islamic finance emerged, there was a need for Islamic corporate governance to follow the rules as per Islamic principles. Corporate governance means to “check and balance a firm’s operations.” It helps to mitigate principal-agent theory, which has long been discussed under corporate governance as it determines the antecedents and compliance with ICG as per the policies set forth by the directors (agents).

Every individual in the market may affect the movements and returns of the market in the least manner. It is beneficial for investors to invest as per the rules of Shariah, because the biggest advantage is risk-sharing, which may lead to the mitigation of losses in most cases.

### Sensitivity of the Islamic Banking Sector

Islamic banking is less sensitive compared to conventional banking, because conventional banking faces the risks of interest rates, whereas, there is no concept of interest rate risk in Islamic banking. Conventional banking has credit risk usually in cash form; on the other hand, Islamic banking has credit risk in the form of assets/products.

Like all other sectors, Islamic banking is not fully efficient in Pakistan, as it is in the growth phase. By contrast, in Malaysia, Islamic banking is efficient as its growth rate is higher than conventional banking. It is significant that non-Muslims also prefer to be part of Islamic banking instead of conventional banking, as they know about the benefit of risk-sharing in any sort of investment.

### The Risk-Taking Propensity of Individuals

Most of the individuals in Pakistan who are aware of Islamic banking must know about the governance mechanism of that place. Those who are unaware, want to learn about it. In Malaysia, almost all the individuals are keen to know about the governance mechanism, as they are open to investing after knowing more about it.

Many individuals in the Islamic banking sector also think they are in a safe position compared to people who are attached to conventional banking, as they are in a position of risk-sharing instead of risk gain or loss on an individual basis.

## Conclusion

According to this study, we can infer that a dearth of independent audit committees and an inefficient corporate governance structure enable fraudulent companies to operate without monitoring activities. For example, if audit committees are dependent and influenced by internal auditors then all the decisions taken create a predilection for self-interest and fraudulent activities.

Keeping the literature in mind we can accept the fact that the gap exists. The process of creating financial instruments means that they should be inimitable and they should not be simply copied from conventional products. They ought to have a competitive edge over conventional finance. The gap also exists in that there is no standardized Islamic finance across borders as there are no sharia standards in national legislation. Some Muslim countries follow Islamic corporate governance models and some of them do not. Take as an example Malaysia, which follows it, and a country such as Saudi Arabia, which is under huge debt and does not abide by the laws of Islam.

## Data Availability Statement

The raw data supporting the conclusions of this article will be made available by the authors, without undue reservation.

## Author Contributions

All authors contributed to this study as per their area of expertise.

## Conflict of Interest

The authors declare that the research was conducted in the absence of any commercial or financial relationships that could be construed as a potential conflict of interest.

## Publisher’s Note

All claims expressed in this article are solely those of the authors and do not necessarily represent those of their affiliated organizations, or those of the publisher, the editors and the reviewers. Any product that may be evaluated in this article, or claim that may be made by its manufacturer, is not guaranteed or endorsed by the publisher.
